# Distance and weightage-based identification of most critical and vulnerable locations of surface water pollution in Kabul river tributaries

**DOI:** 10.1038/s41598-023-38018-8

**Published:** 2023-07-18

**Authors:** Muhammad Irfan, M. Mahboob Alam, Shahbaz Khan, Ilyas Khan, Sayed M. Eldin

**Affiliations:** 1 Department of Civil Engineering, University of Engineering and Applied Science, Swat, 19060 Pakistan; 2 Department of Cıvil Engineering, City University of Science and Information Technology, Peshawar, Pakistan; 3 Department of Mechatronics Engineering, University of Engineering and Applied Science, Swat, 19060 Pakistan; 4 Department of Mathematics, College of Science Al-Zulfi, Majmaah University, Al-Majmaah, 11952 Saudi Arabia; 5 Center of Research, Faculty of Engineering, Future University in Egypt, New Cairo, 11835 Egypt

**Keywords:** Environmental sciences, Engineering

## Abstract

Water plays a key role in the economic growth of an agricultural country. Pakistan is a farming country that uses almost 90% of its water resources for agriculture. Khyber Pakhtunkhwa (KPK) province of Pakistan has extensive surface water resources. In addition to using groundwater resources for irrigation, large parts of its flat plains are irrigated with the Kabul River surface water. Due to large population growth and unregulated small/local scale industries in the region, surface water quality deteriorates with time, which affects people's health when polluted surface water is used for irrigation purposes. This research investigates the surface water quality of Kabul River's different tributaries. It identifies the most critical and vulnerable locations regarding water quality using the weightage-based identification method and distance-based iteration method, respectively. The Bara River exhibited the most critical location, surpassing the threshold values by a considerable margin in at least seven water quality parameters. The maximum seven critical values determined against the Bara River using the weightage-based method, i.e., 17.5, 5.95, 7.35, 27.65, 1.75, 0.35, and 10.45 for total alkalinity, sodium, total hardness, magnesium, total suspended solids, biological oxygen demand (BOD), and turbidity. The Khairabad station, where the Kabul River meets the Indus River, was identified as vulnerable due to elevated levels of total suspended solids, hardness, sulfate, sodium, and magnesium using distance-based methods. The locations, i.e. Adezai, Jindi, Pabbi, and Warsak Dam, appeared critical and vulnerable due to the prevalence of small-scale industries on their bank and high population densities. All the results are finally compared with the interpolated values over the entire region using Kriging interpolation to identify critical and vulnerable areas accurately. The results from the distance and weightage-based methods aligned with the physical reality on the ground further validate the results. The critical and vulnerable locations required immediate attention and preventive measures to address the deteriorating water quality parameters by installing monitoring stations and treatment plants to stop further contamination of the particular parameter.

## Introduction

Climate characterizes the long-term average weather conditions in a particular location, encompassing variations, extremes, and changes over time. On the other hand, climate change refers to a statistically significant and persistent alteration in the mean state of the climate over an extended period, which can be influenced by internal processes within the climate system and external factors such as changes in the atmosphere or land use. Social migration can impact local immigrant communities, but it is important to note that climate change refers to changes in the climate system and not directly to social mechanisms. Climate variability encompasses natural internal processes within the climate system and variations caused by natural or human-induced external forces. The world has recently faced a concerning trend of increasing temperatures at an average rate of 0.128 ± 0.026 °C per year over 59 years^1^. Precipitation plays a significant role in influencing soil moisture levels in unplanned economic activities. The moisture content of soils varies depending on factors such as soil type, season, and the amount of rainfall received. A study observed that Rainwater Harvesting (RWH) led to notable increases in soil moisture, with a 59% increase from the Surface to a depth of 15 cm, a 63% increase at depths ranging from 15 to 30 cm, and an 80% increase in the 30–45 cm depth. These findings indicate that RWH effectively enhances soil moisture across different conditions worldwide^2^. 

The regions of South Asia will perhaps become dry after 2025, negatively impacting agricultural practices and water requirements and causing a decrease in the crop yield of about 6–18% in arid and semi-arid areas. Managing surface water resources and an area's physiographic traits is important for humans and practising agriculture. Considering its importance in agriculture; ecosystem activities and agricultural planning have been considered a vital sector in irrigation planning by policymakers worldwide. The dry and wet season is considered the basic factor for climatic change in Asian regions of Pakistan, including India and Pakistan. Droughts are one of the multidimensional extreme events that negatively influence agriculture, land use, water availability, and food security in the World^3^. The hydrological response resulting from climate change may significantly impact existing water resource systems by changing the hydrological cycle. Therefore, hydrologic extremes like floods and droughts negatively impact all watersheds^4^. However, planning and organizing the new water infrastructure are not developed according to the projected changes in precipitation, temperature, and streamflows for efficient water resource management^5^.

Water is a limited and fundamental resource for life, exchanging its form in a continuous cycle between land and atmosphere^6^. While the classification of available water is done in multiple ways, the most common classification is based on the availability of water relative to its source, especially surface water, groundwater, and atmospheric water. Without going into the forms in which water is available and used in ground and atmospheric sources, we look into surface water and its use for producing agricultural products. Within the terrestrial ecosystem, the same water used for crop production is shared with the public and aquatic life^7^. Surface water classifications are applied to surface water bodies, such as streams, rivers, and lakes. The general use of these waters is for drinking water supply, irrigation, fishing, industrial use, transport, and recreation^8^. In Pakistan, surface water resources are predominantly used for agriculture. Agriculture is key to Pakistan's gross domestic product (GDP). According to the Economic Survey of Pakistan 2022, the Agriculture sector contributes about 22.7% to the GDP, which employs 37.4% of the labor force and 64% of export earnings. It provides livelihoods to 62% of the country's population^9^. Regarding water resources, agriculture is one of the biggest consumers, using over 90% of available fresh water in Pakistan^10^.

The heavy burden on agricultural produce for national consumption and for enhancing foreign exchange reserves through exports mandates the growers to use large amounts of fertilizers to supplement the naturally available nutrients in the soil which ultimately affects the groundwater quality in the long run^11^. Numerous studies have established the causal relations between the use rate of fertilizers and deterioration in groundwater quality over time^12,13^. Unsustainable water use, whether groundwater or surface water, also ultimately affects groundwater quality. The connecting interface of groundwater and surface water makes it almost impossible not to affect one resource while the other is exploited^14^.

Draining untreated or partially treated water from industries, hospitals, and other public and commercial buildings into surface water bodies is another major source of pollution in surface water^15^. While in the developed world, strict regulations ensure water treatment before it can be drained into surface water bodies, the same may exist on paper. However, enforcement of these important regulations is usually found wanting^16^. In a recent case, the high court bench of a city in Pakistan summoned relevant government authorities regarding the disposal of effluents from different kinds of industries directly into surface water bodies. The industries pointed out were construction material-producing, ranging from cement to steel reinforcements, pharmaceutical, and paper industries. There is also evidence of shallow groundwater contamination in India from industries of bicycles and ancillary parts, electroplating, steel, and foundries resulting in a high concentration of cyanide in groundwater^17^. The largest source of surface water pollution in Pakistan is municipal wastewater, which pollutes rivers, drains, and streams downstream of large cities with organic matter, suspended solids, and surfactants^18^. Industrial wastewater discharges are also high, polluting surface waters with heavy metals, oil products, phenols, and other hazardous substances^11,19^.

In the case of Pakistan, the problem is not only with the quality of water but also the available water quantity. Based on generally accepted thresholds of available water in a country on per capita and per annum basis^20^, Pakistan touched the "water stress line" in 1990 and crossed the "water scarcity line" in 2005. If the population growth and water consumption rates by different economic sectors remain unchanged, the country may run dry by 2025^21^. The surface water resources of Pakistan are mainly based on the flows of the Indus River and its tributaries^22^. The Indus River is 2900 kms, and the drainage area is about 966,000 sq. km. Five major tributaries join it from the eastern side, and three minor tributaries drain in the mountain region. Several small tributaries also join the Indus River towards its western side. One of the biggest tributaries of the Indus River is River Kabul^23^.

**Figure 1:**
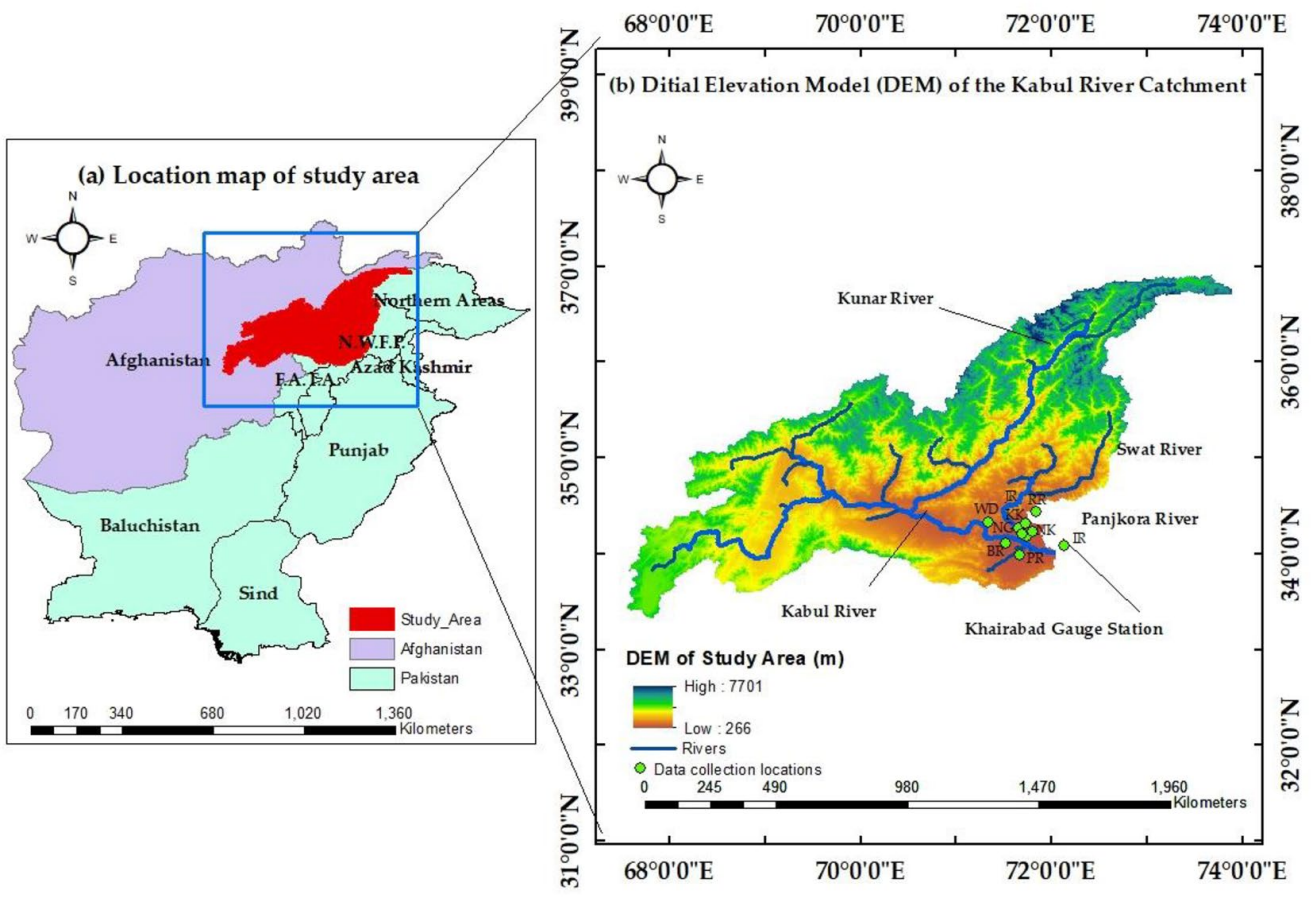
Location map of (**a**) Pakistan and Afghanistan (**b**) the whole Kabul River basin's digital elevation model.

Kabul River can be seen in Fig. 1, which is the western tributary of the Indus River, contributes 16.5 million acre-feet (MAF) of water to Pakistan's complex water system^24^. Before flowing into KPK province in Pakistan, the Kabul River passes through Kabul, Surobi, and Jalalabad in Afghanistan, 25 km (16 Miles) north of the Durand line. In KPK province, the Kabul River passes through Peshawar, Charsadda, and Nowshera. Its largest tributary is the Kunar River, which starts as the Mastuj River, flowing from the Chiantar glacier in Brughil Valley in Chitral, Pakistan, and after flowing south into Afghanistan, joins the Bashgal River flowing from Nurestan, eastern Afghanistan^25^.

In River Kabul, about 72% of the runoff occurs between May and September, and 28% occurs between October to April. The peak runoff is generally in June and July. In comparison, the peak demand for agriculture is during July and August^26^. The Kabul River and its tributaries transport untreated sewage from Afghanistan and Pakistan's cities, towns, and villages from Peshawar, Mardan, Khyber, Mohmand, and Malakand agencies^27^. The lower sections of the river pass through the plains, particularly densely populated. The effluents from many industries end up in the river Kabul, either directly or through nullahs which eventually drain into the river. Of these industries, the sugar mills, distilleries, paper mills, tanneries, ghee factories, and textile mills contribute most of the water pollution hazards^28^.

The wastewater contains domestic, industrial, and commercial effluents and their quality is checked using a variety of laboratory tests, including tests for physical, chemical, and biological parameters^29^. Further research has been done to analyze the water quality of a region based on these tests using conventional techniques. It highlights the common risks and possible suitable solutions in water supply for domestic and agricultural use^30^. The surface and groundwater quality of the Kabul River at Attock City, Punjab, has been investigated by calculating the water quality index (WQI)^31^. The status of groundwater was compared with World Health Organization (WHO) standards and found a negative trend in nitrates and faecal microbes in the Kabul catchment^32^. Another research that has been done to investigate the concentration of heavy metals in the surface water of the Kabul River found that nickel has the highest concentration, 30 times higher than the permissible limit of WHO^33^. This research idea was used to identify the flood risk based on the most crucial variables of flood characteristics of Waverly City, Iowa, United States^34^.

Pakistan Council of Research in Water Resources (PCRWR) studied water quality parameters of the upper Khyber Pakhtunkhwa (KPK) region and northern areas of Pakistan including Mardan, Buner, and Swat districts of KPK, among various other regions^35,36^. Although the specific districts focused on this study were not in the PCRWR study, the results exhibit similarities with the findings of the PCRWR study conducted in district Mardan. District Mardan, which shares geographical proximity with the locations of this study, also exhibits similar geological, social, and industrial conditions. According to the International Union for Conservation of Nature, Pakistan Program (IUCN), the Kabul watershed houses 205, 10, 41, and 45 industrial units in Peshawar, Charsadda, Nowshera, and Mardan districts, respectively, constituting more than 15% of the total industries in the region^37^. Almost all these industries have no treatment facilities for effluents. Nitrites from agricultural fertilizers and untreated effluents like ammonia, chromium, and nickel from industries influence the water quality in these areas^38^.

The research conducted on the water quality status of the Kabul River and its tributaries stands out as a valuable and distinctive contribution in several ways. Unlike previous studies that solely relied on the water quality index, this research goes beyond the surface level by thoroughly investigating the water quality of all tributaries connected to the Kabul River. By considering the water quality status of these tributaries, the study adopts a comprehensive approach that combines weightage and distance methods to identify the most critical and vulnerable location. Additionally, the research utilizes interpolated maps in arc-GIS to compare and analyze the results spatially, providing a deeper understanding of the water quality dynamics. This study identifies critical tributaries and proposes practical and effective solutions for water quality issues. These solutions include the installation of monitoring stations on the identified tributaries and recommendation-specific treatment procedures to mitigate the long-lasting effects of water contamination.

Moreover, the research emphasizes the importance of stakeholder involvement and advocates for launching awareness campaigns, highlighting the need for collective action in vulnerable locations. The uniqueness of this study lies in its comprehensive approach, methodological framework, and practical recommendations, which collectively provide valuable insights and guidance to concerned authorities and decision-makers. This study becomes an invaluable resource for future research by facilitating the implementation of remedial measures. It contributes to protecting and preserving the Kabul River and its tributaries, ensuring the well-being of the communities and the environment that depend on these water resources. Section 3 describes the detailed problem solution with research findings, and Sect. 4 discussed the study's conclusion.

## Material and methods

### Study area and data collection

The stretch of the Kabul River under study is the section from just upstream of Warsak Dam to its junction with the Indus. This area is densely populated, with much of the KPK's small industry dependent upon the water of the Kabul River and its tributaries for different purposes^35,39^. Most industries ultimately drain their effluents without any treatment in the Kabul River. Ironically, this is also where much of the agricultural products consumed in cities like Peshawar, Mardan, and Swat are grown. Much concern has been raised about the quality of water in these areas^31^. The geographical boundary of the Kabul River basin and the sampling locations are shown in Fig. 1. The sampling points are shown in green dots and a detailed explanation of the sampling locations is mentioned in Table 1.

**Table 1:** Sampling points details of the study area.

Sampling points	Latitude	Longitude	Altitude (ft.)	River ID
Warsak Dam	34.16442	71.357898	1257	WD
Adezai River	34.08889	71.74889	951.5	AK
Naghuman River	34.12169	71.607621	1005	NG
Khiyali River	34.12194	71.70889	961.04	KK
Jindi River	34.15629	71.734471	977	JR
Bara River	33.9668	71.561619	1171	BR
Pabbi River	33.97382	71.748456	1026	PR
Nowshera Kallan River	34.00887	71.985346	930	NK
Rustam River	34.34967	72.278996	1233	RR
Indus River at Khairabad	33.89687	72.233806	875	IR

All the water samples were collected from ten different tributaries in 1-L polyethene (PE) bottles, which were washed with deionized water before use that drained into the Kabul River. These sample bottles were sealed and placed in a dark environment at a constant temperature range of 4–10 °C to avoid contamination and the effects of light and temperature. The detail of the sampling locations with a unique ID was the tributary of Kabul River along with their latitude and longitude and their height from the mean sea level given in Table 1.

All standard protocols necessary for sample collection, sample storage, sample transportation, and sample tests were followed as per relevant American Society of Testing and Materials (ASTM) guidelines given in Table 2. A total of nine parameters were tested on the collected water quality sample under the relevant standards mentioned in the parameter section, followed by the units of each parameter, their accuracy, test standards and Food and Agriculture Organization (FAO) and WHO threshold values both for drinking and agriculture use.

**Table 2:** Water quality test standards used along with Apparatus used and threshold values of WHO and FAO.

Parameters	Units	Apparatus	Accuracy	Test standard	WHO drinking water limits	FAO limits for agricultural use
PH		PH meter with Buffer solution	± 0.002	ASTM D1293-99	6.5–8.5	5–8.5
Color	TCU	Multi-parameter Photometer		ASTM D1209-05	5	Unpleasant/Acceptable
Taste and Odor		Acceptable/Non-Acceptable		ASTM E2892-15	Acceptable	Unpleasant/Acceptable
Total Suspended Solids (TSS)	mg/L	Filter paper, beaker, Desiccators, titration assembly, vacuum pump, and conical flask	± 0.5%	ASTM D5907-18	25	120
Total Dissolved Solids (TDS)	mg/L	Filter paper, beaker, Desiccators, titration assembly, vacuum pump, and conical flask	± 0.5%	ASTM D5907-10	300	0–2000
Electric Conductivity (EC)	µs/cm	DDS-22C High Accuracy Digital Conductivity meter	± 1.0%	ASTM D1225-14	400	> 3
Dissolved Oxygen (DO)	mg/L	BOD bottles and other related chemicals	± 1.0%	ASTM D888-09	6.5–8	5.0–8.0
Biochemical Oxygen Demand (BOD)	mg/L	BOD bottles and other related chemicals	± 1.0%	ASTM D2329-68	0	80
Chemical Oxygen Demand (COD)	mg/L	BOD bottles and other related chemicals	± 1.0%	ASTM D1252-06	< 250	150

The samples were analyzed for their physical, chemical, and biological properties relevant to the study. Considering the pollution sources the study area is exposed to and the ultimate use of untreated surface water for irrigation purposes, physical parameters like pH, Total Settleable/Suspended Solids (TSS), Total Dissolved Solids (TDS), Turbidity, and Electrical Conductivity (EC)^40^ were measured. For chemical analysis, water samples were tested for Total Alkalinity (TA), Sodium (Na), Calcium (Ca), Magnesium (Mg), Total Hardness, Nitrite (NO_2_^−^), Sulfate (SO_4_^2−^), Phosphate (PO_4_), Chloride (Cl^-^), and Arsenic (As)^41^. Biochemical Oxygen Demand (BOD), Chemical Oxygen Demand (COD) and Dissolved Oxygen (DO)^42^ limits were measured for biological properties.

### Distance and weightage-based identification of most critical and vulnerable locations

**Figure 2:**
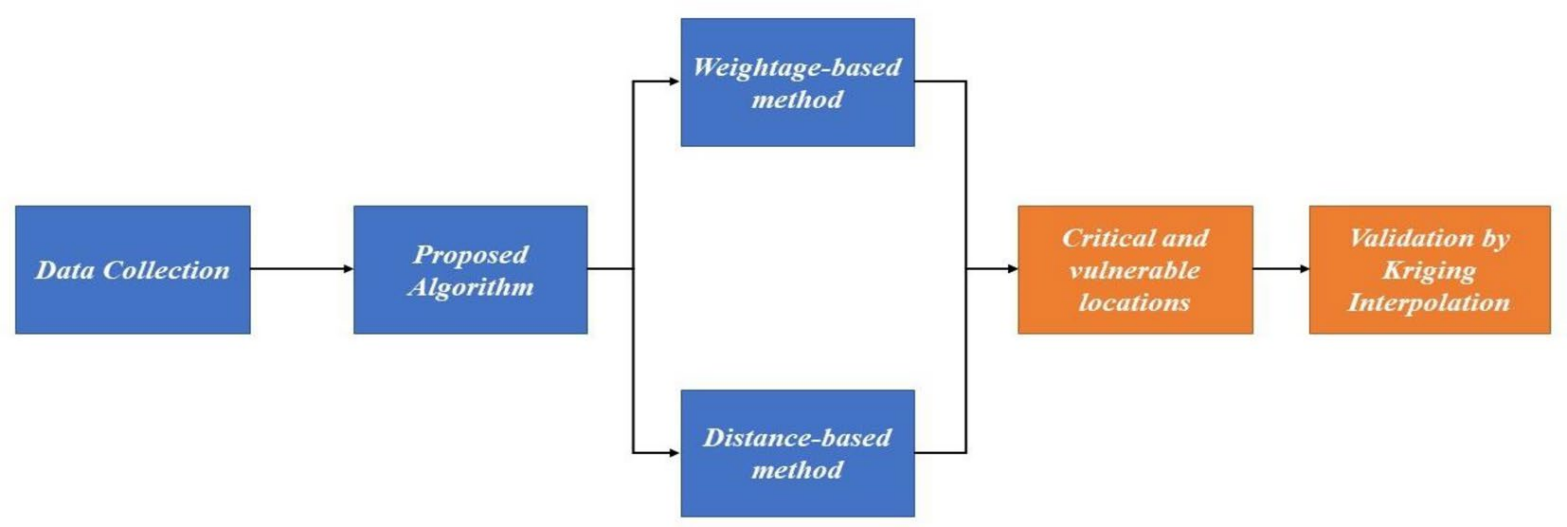
Illustration of the adopted methodology in identifying critical and vulnerable locations.

The methodology presented here is developed to identify the most critical and vulnerable locations having polluted groundwater. We define the criticality of a certain location based on a large value exceeding the threshold defined by WHO. This way, we differentiate between the locations with observed parameter values just exceeding the threshold amount and those exceeding the threshold by a large margin. We define the vulnerability of certain locations by identifying areas closest to critical areas identified earlier in terms of their observed values. An iterative distance-based indicator is used to identify locations that need immediate intervention to secure water quality. The detailed procedure used in this paper is illustrated in a flow chart shown in Fig. 2.

#### Critical location identification

Mathematical equations identify the critical location with the help of certain observed parameters, i.e., physical, chemical, and biological parameters. The threshold value is escalated by 50% and added to the original threshold value given by WHO as given in Eq. (1). Then compare the measured values at all locations with the escalated values instead of the original threshold values to find the severity of exceedance using Eq. (4).

*Proposed Algorithm of Weightage-based method*

Indices

- es = escalated values by 50%
- i = location of sample collection
- j = recorded value of water quality parameter
- ex = exceedance value (means near to exceeding the threshold value)

Parameters

- ${PP}_{j}^{es}$: water quality measured value *j* of physical parameter *PP* escalated by 50% *es*.
- ${CP}_{j}^{es}$ : water quality measured value *j* of chemical parameter *CP* escalated by 50% *es.*
- ${BP}_{j}^{es}$ : water quality measured value *j* of biological parameter *BP* escalated by 50% *es*.
- ${PP}_{who,j}$: threshold value set by *WHO* for physical parameter *PP*
- ${CP}_{who,j}$: threshold value set by *WHO* for chemical parameter *CP*
- ${BP}_{who,j}$: threshold value set by *WHO* for biological parameter *BP*
- ${PP}_{ij}$: observed values *j* of physical parameter *PP* for particular location *i*
- ${CP}_{ij}$: observed values *j* of chemical parameter *CP* for particular location *i*
- ${Z}_{ij}$: exceedance threshold value *Z* for particular location *i* of sub parameter *j*
- ${II}_{ij}$: importance indicator *II* for particular location *i* of sub-parameter *j*
- ${CL}_{j}$: critical location *CL* based on sub-parameter *j*
- ${PP}^{ex}$: exceedance value *ex* of physical parameter *PP*
- ${VL}_{j}$: vulnerable location *VL* based on sub-parameter *j*

For a particular physical parameter, the escalated threshold value would be:

1 $${PP}_{j}^{es}= \left(0.5* {PP}_{who,j}\right)+{PP}_{who,j}$$

Similarly, Eqs. (2) and (3) used for chemical and biological parameters:

2 $${CP}_{j}^{es}= \left(0.5* {CP}_{who,j}\right)+{CP}_{who,j}$$

3 $${BP}_{j}^{es}= \left(0.5* {BP}_{who,j}\right)+{BP}_{who,j}$$

Then Eq. (4) is used to calculate the extent to which certain water quality parameter exceeds the threshold *Z* for all the water quality parameters, i.e.,

4 $${Z}_{ij}={PP}_{ij}-{PP}_{who,j}$$

Moreover, compare it with the escalated threshold values as defined above.

Depending on whether Z exceeds the escalated threshold at a certain location, weights are assigned to these locations to single them out among all locations. The importance indicator (*II*) for a certain location thus gives us an idea of the extent of intervention needed to mitigate the exceedance situation at a particular location.

5 $${II}_{ij}=\left[\begin{array}{c}\begin{array}{c}0.9* {Z}_{ij}\,\, if {Z}_{ij}>{PP}_{j}^{es}\\ \end{array}\\ 0.35* {Z}_{ij}\,\, if {Z}_{ij}< {PP}_{j}^{es}\end{array}\right]$$

Finally singled out the critical location for a particular water quality parameter *j* by choosing the location with has the maximum *II* value.

6 $${CL}_{j}=\underset{i\in \left[1..n\right],j}{\mathrm{max}}{II}_{ij}$$

where *n* is the total number of locations over which the search for maximum is performed.

The above methodology would work for those water quality parameters for which the desired value does not exceed the upper threshold value defined by a regulatory body (e.g., Total Hardness). In case the desired value is higher than the defined threshold (Dissolved Oxygen) value, the minimum value among all locations will be used, i.e.

7 $${CL}_{j}=\underset{i\in \left[1..n\right],j}{\mathrm{min}}{II}_{ij}$$

Critical locations obtained for each water quality parameter thus identify locations that exceed the threshold values by a large amount. Water use from these areas should be strictly regulated, which is especially important for irrigation water use to avoid harmful chemicals entering the food chain.

#### Vulnerable Location Identification

This study proposes an iterative distance-based search method to identify the most vulnerable locations (VL) in terms of exceedance of threshold values. The method is used for locations that, though not yet exceeding the threshold limit, are most likely to do so shortly based on the observed value.

*Proposed Algorithm of the distance-based method*

8 $${VL}_{j}=\mathrm{min}\left(abs\left|{PP}_{ij}-{PP}^{ex}\right|\right)$$

9 $${VL}_{j}=\mathrm{min}\left(abs\left|{CP}_{ij}-{CP}^{ex}\right|\right)$$

Equations (8) and (9) inherently show the methodology's iterative nature. We search for locations where the observed values of different physical, chemical, and biological parameters are the closest to the exceeding value. The results would benefit the decision-makers in prioritizing the mitigating measure for a particular location.

*Validation by Kriging interpolation* To compare the results obtained from the above methodology, we employ the Kriging interpolation method in our study area for identifying the most critical locations. Using a 30-m digital elevation model of the region as external drift, we interpolate the observed values of different parameters over the entire region.

### Laboratory test results

**Figure 3:**
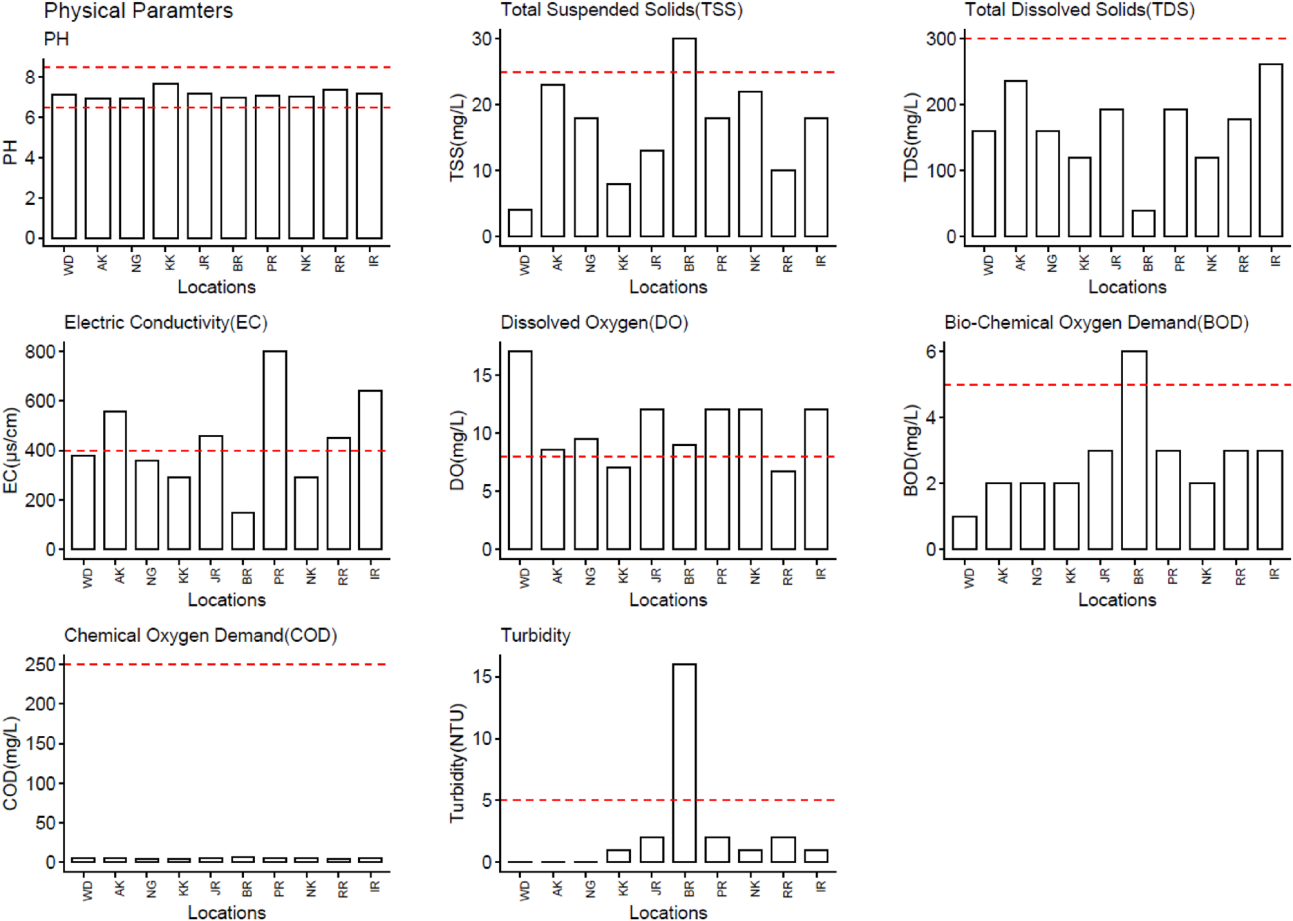
Results of Physical water quality tests performed at each location.

**Figure 4:**
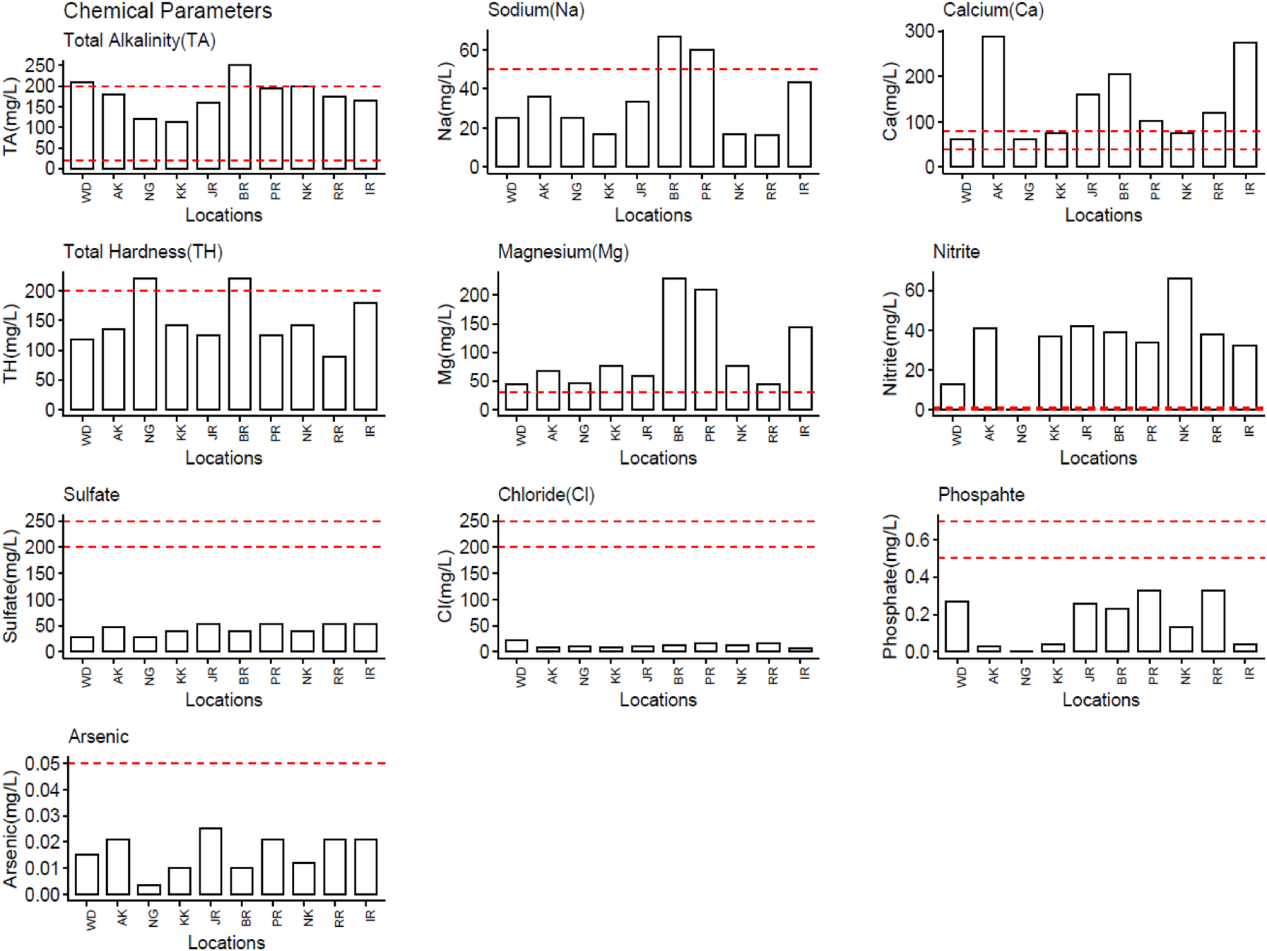
Results of Chemical water quality tests performed at each location.

**Figure 5:**
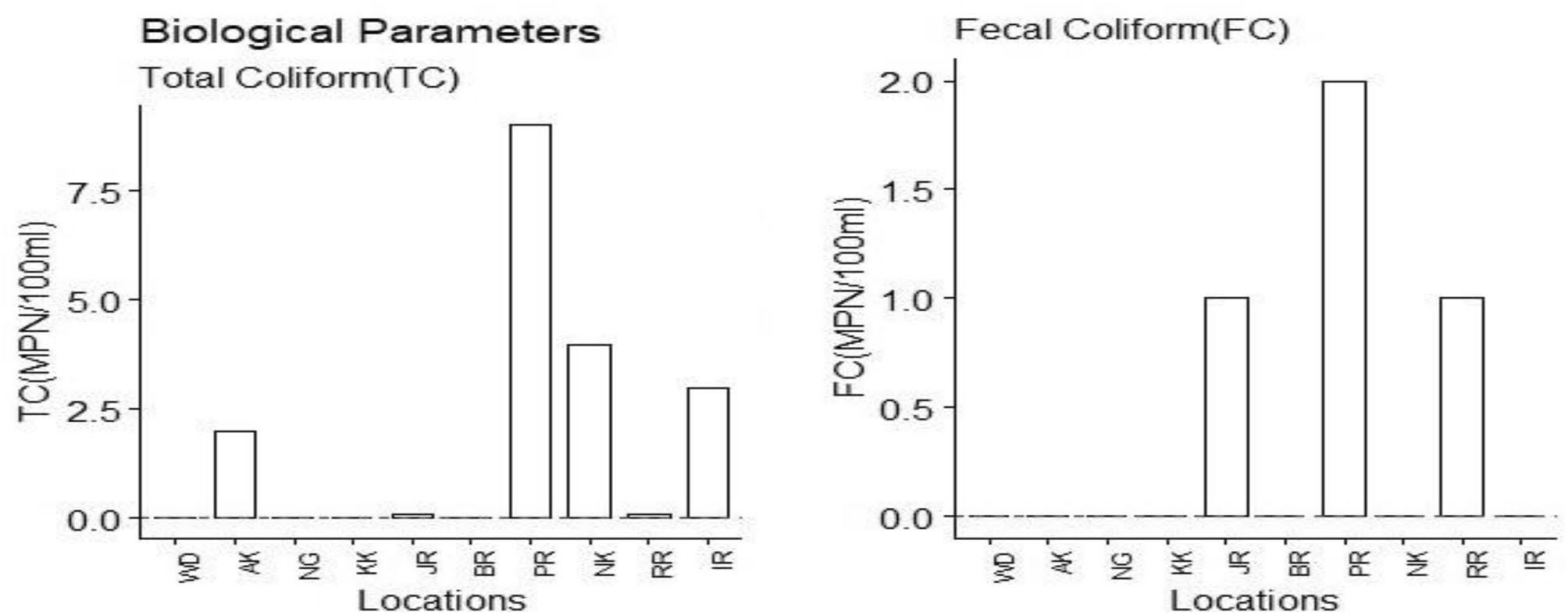
Results of Biological water quality tests performed at each location.

Figures 3, 4, and 5 show the summary results of water quality tests of selected physical, chemical, and biological parameters. The WHO-specified allowable/threshold limits, used as guiding limits, are depicted as red horizontal lines for each parameter. Threshold limits are "triggers" for starting mitigating efforts for bringing the recorded values to safe values and an "endpoint" for terminating mitigating efforts.

The overall test results contradict the previous general findings about water quality in these areas. Except for EC and DO, almost all considered physical parameters lie below the threshold limits. The purer the water, the lower the conductivity. Distilled water is almost an insulator, while salty water is an electrical conductor. Half of the selected locations exceed the threshold limits of EC. Almost all locations exceed the DO values by a considerable margin. Surface water having higher DO values (or too low) affect surface water's aquatic life and its quality.

During chemical analysis of water quality parameters (Fig. 3), selected locations show mixed results. Ca, Mg, and NO_2_ exceeded threshold values in almost all locations. Samples taken from Bara River (BR) seem to have the worst surface water quality in terms of both physical (TSS, BOD, Turbidity) and chemical (Na, TH, TA) water quality parameters and it observes that the water quality parameters measured in the samples taken from Bara River (BR) deviate from the patterns observed in other locations. These deviations indicate that Bara River exhibits distinct characteristics or trends compared to the other sampled locations. The reason could be that most of the small-scale industry at this location (Marble and Paper industry) drains its effluents directly into the surface water streams. This makes the surface water at this location increasingly polluted concerning the quality parameters where other locations have reasonably acceptable values.

**Figure 6:**
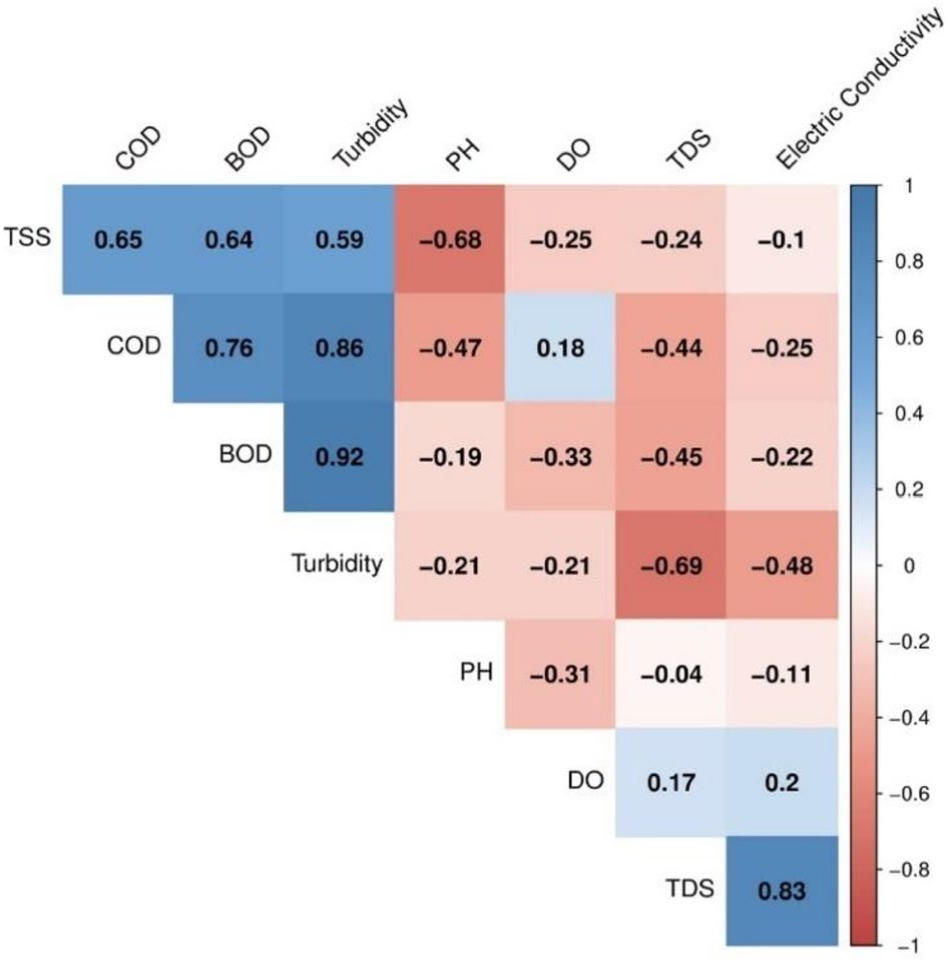
Pearson correlation values between physical water quality parameters at all locations.

Figure 6, e.g., show the selected location results for biological parameters such as total and faecal coliforms. All considered locations exceed the acceptable threshold limits of WHO for these parameters. Malik et al. (2010) refer to the WHO study, which says that faecal bacteria, parasites, and other microbes cause about 6000 deaths of adults and children every day, resulting in a statistic of 1.8 million deaths every year from complications caused by the presence of this kind of pollutant in water bodies^43^.

**Figure 7:**
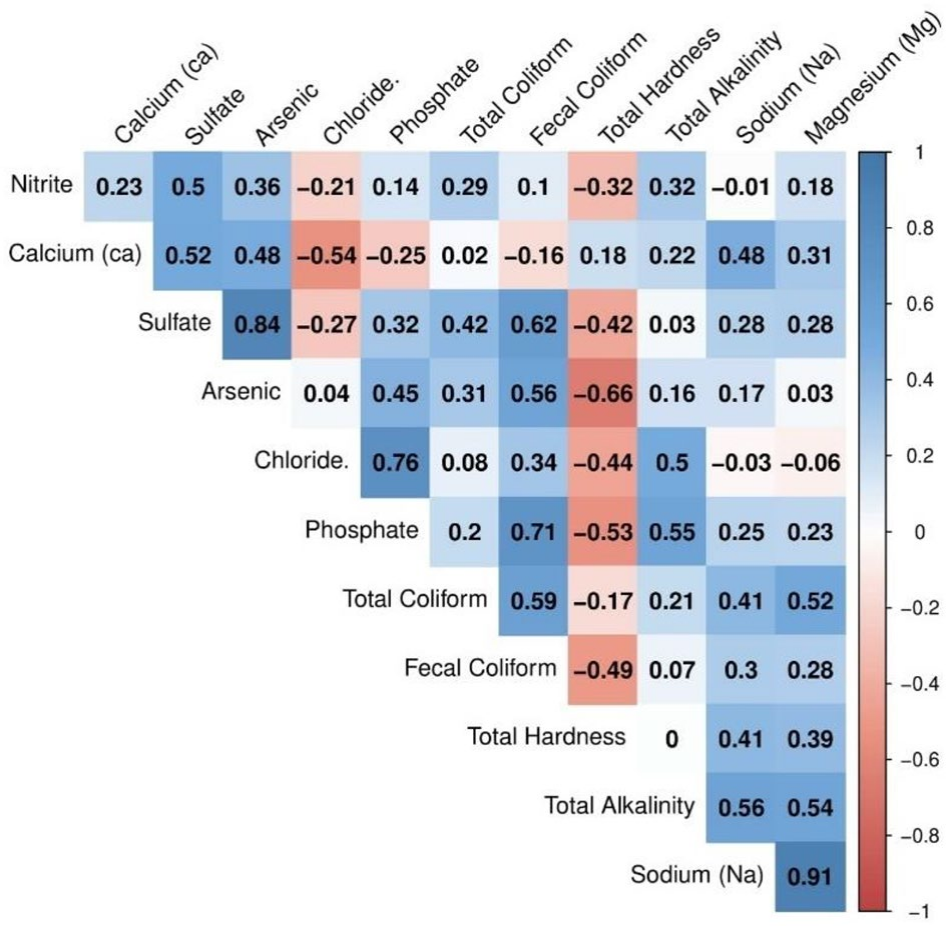
Pearson correlation values between chemical/biological water quality parameters.

Figures 6 and 7 shows a correlation between measured physical and chemical-biological water quality parameters. These correlations were calculated to ensure the samples were properly collected, stored, transported, and tested. According to our understanding of the dependence of different water quality parameters on each other and their confirmation from the correlation test gives us confidence that the data collected is trustworthy. For example, the main salts generally dissolved in water are carbonates, bicarbonates, sulfates, chlorides, nitrates, and phosphates. The presence of TDS in water is commonly associated with a higher probability of increased electric conductivity. The correlation between TDS and EC also provides confidence in the reliability of the collected data, reinforcing the interdependence of various water quality parameters^44^. We obtain a high Pearson correlation value between TDS and EC of 0.83 (Fig. 6). Similarly, high correlation values were obtained between measured TSS and parameters like Turbidity, TH, and COD. The Turbidity/COD and Turbidity/BOD similarly display correlation values above 0.85.

## The research findings

Mathematical Eqs. (1)–(3) were applied to escalate the threshold value by 50% and the threshold value set by WHO. In Table 3, the first line of each parameter is the observed value denoted by CP, PP and BP. In contrast, the second line is the escalated value addition with the WHO values of each parameter for all locations. All the exceeded values were highlighted as bold; the bold, positive values indicate that the observed values exceeded the except for dissolved oxygen. The count of highlighted values in all water quality parameters for BR, PR, RR, JR, IR, and AK are 8, 7, 6, 5, and 4, respectively, indicating that BR repeats the most, followed by PR. This indication will be further confirmed by applying the remaining equations.

**Table 3:** Measured values are represented as CP/BP/PP, while the escalated values are as Z.

Parameter	Terms	WD	AK	NG	KK	JR	BR	PR	NK	RR	IR
Total alkalinity	${CP}_{ij}$	210	180	120	114	160	250	195	200	175	165
	${Z}_{ij}$	***10***	− 20	− 80	− 86	− 40	***50***	− 5	***0***	− 25	− 35
Sodium	${CP}_{ij}$	25.2	36.3	25.1	17.1	33.5	67	60	17.1	16.45	43.5
	${Z}_{ij}$	− 24.8	− 13.7	− 24.9	− 32.9	− 16.5	***17***	***10***	− 32.9	− 33.5	− 6.5
Calcium	${CP}_{ij}$	62	289	62.5	76	160	206	101	76	120	275
	${Z}_{ij}$	− 18	***209***	− 17.5	− 4	***80***	***126***	***21***	− 4	***40***	***195***
Total hardness	${CP}_{ij}$	118	136	221	143	125	221	125	143	89	180
	${Z}_{ij}$	− 82	− 64	***21***	− 57	− 75	***21***	− 75	− 57	− 111	− 20
Magnesium	${CP}_{ij}$	45	67	45.2	77	58	229	210	77	44.45	143
	${Z}_{ij}$	− 105	− 83	− 104	− 73	− 92	***79***	***60***	− 73	− 105.5	− 7
Nitrite	${CP}_{ij}$	13	41	0	37	42	39	34	66	38	32
	${Z}_{ij}$	***12***	***40***	− 1	***36***	***41***	***38***	***33***	***65***	***37***	***31***
Total coliform	${BP}_{ij}$	0	2	0	0	0.1	0	9	4	0.1	3
	${Z}_{ij}$	0	***2***	0	0	***0.1***	0	***9***	***4***	***0.1***	***3***
Fecal coliform	${BP}_{ij}$	0	0	0	0	1	0	2	0	1	0
	${Z}_{ij}$	0	0	0	0	***1***	0	***2***	0	***1***	0
Total suspended solids	${PP}_{ij}$	4	23	18	8	13	30	18	22	10	18
	${Z}_{ij}$	− 21	− 2	− 7	− 17	− 12	***5***	− 7	− 3	-15	− 7
Electric conductivity	${PP}_{ij}$	380	560	360	290	460	150	800	290	450	640
	${Z}_{ij}$	− 20	***160***	− 40	− 110	***60***	− 250	***400***	− 110	***50***	***240***
Dissolved oxygen	${PP}_{ij}$	17	8.6	9.5	7	12	9	12	12	6.7	12
	${Z}_{ij}$	9	0.6	1.5	− ***1***	4	1	4	4	− ***1.3***	4
Biochemical oxygen demand	${PP}_{ij}$	1	2	2	2	3	6	3	2	3	3
	${Z}_{ij}$	− 4	− 3	− 3	− 3	− 2	***1***	− 2	− 3	− 2	− 2
Turbidity	${PP}_{ij}$	0	0	0	1	2	16	2	1	2	1
	${Z}_{ij}$	− 5	− 5	− 5	− 4	− 3	***11***	− 3	− 4	− 3	− 4

The exceeded values (${Z}_{ij}$) were assigned special weightage of 90% and 35% by following the condition of Eq. (5) and obtained the importance indicator values (II) for all the locations using the values of Table 3. All the highlighted values in Table 4 are the critical values obtained using Eq. (6).

**Table 4:** The highlighted values are the Critical values using the Eqs. (6 and 7).

Location	WD	AK	NG	KK	JR	BR	PR	NK	RR	IR
Total alkalinity	3.5	− 7	− 28	− 30.1	− 14	***17.5***	− 1.75	0	− 8.75	− 12.25
Sodium	− 8.68	− 4.795	− 8.715	− 11.515	− 5.775	***5.95***	3.5	− 11.515	− 11.74	− 2.27
Calcium	− 6.3	***188.1***	− 6.125	− 1.4	28	113.4	7.35	− 1.4	14	175.5
Total hardness	− 28.7	− 22.4	7.35	− 19.95	− 26.25	***7.35***	− 26.25	− 19.95	− 38.85	− 7
Magnesium	− 36.75	− 29.05	− 36.68	− 25.55	− 32.2	***27.65***	21	− 25.55	− 36.94	− 2.45
Nitrite	10.8	36	− 0.35	32.4	36.9	34.2	29.7	***58.5***	33.3	27.9
Total coliform	0	1.8	0	0	0.09	0	***8.1***	3.6	0.09	2.7
Fecal coliform	0	0	0	0	0.9	0	***1.8***	0	0.9	0
TSS	− 7.35	− 0.7	− 2.45	− 5.95	− 4.2	***1.75***	− 2.45	− 1.05	− 5.25	− 2.45
Electric conductivity	− 7	56	− 14	− 38.5	21	− 87.5	***140***	− 38.5	17.5	84
DO	***3.15***	0.21	0.525	− 0.35	1.4	0.35	1.4	1.4	− 0.455	1.4
BOD	− 1.4	− 1.05	− 1.05	− 1.05	− 0.7	***0.35***	− 0.7	− 1.05	− 0.7	− 0.7
Turbidity	− 1.75	− 1.75	− 1.75	− 1.4	− 1.05	***10.45***	− 1.05	− 1.4	− 1.05	− 1.4

Table 5 shows all the vulnerable values identified by the distance-based iterative objective function using Eqs. (7) and (8). All the observed values were subtracted from the WHO standard values except the highlighted value in Table 3. The values that exceeded the observed values were already used for identifying critical locations, therefore not given in Table 5, and left as an empty box. The minimum value in each row is the most vulnerable, and the location is the most vulnerable.

**Table 5:** The highlighted values are vulnerable values using Eqs. (8 and 9).

Location	WD	AK	NG	KK	JR	BR	PR	NK	RR	IR	VL
Total alkalinity	−	20	80	86	40	−	5	−	25	35	**5**
Sodium	24.8	13.7	24.9	32.9	16.5	−	−	32.9	33.55	6.5	**6.5**
Calcium	18	−	17.5	4	−	−	−	4	−	−	**4**
Total hardness	82	64	−	57	75	−	75	57	111	20	**20**
Magnesium	105	83	104.8	73	92	−	−	73	105.55	7	**7**
Nitrite	−	−	1	−	−	−	−	−	−	−	**1**
Sulfate	221.86	202.84	222.09	210.38	196.03	210.38	196.03	210.38	196.03	196.03	**196.03**
Chloride	227	241	239	242	239	237	233	237	233	244	**227**
Phosphate	0.43	0.67	0.7	0.66	0.44	0.47	0.37	0.57	0.37	0.66	**0.37**
Arsenic	0.035	0.029	0.046	0.04	0.025	0.04	0.029	0.038	0.029	0.029	**0.025**
TSS	21	2	7	17	12	−	7	3	15	7	**2**
TDS	140	64	140	180	107	261	107	180	122	39	**39**
Electric conductivity	20	−	40	110	−	250	−	110	−	−	**20**
DO	9	0.6	1.5	−	4	1	4	4	−	4	**0.6**
BOD	4	3	3	3	2	−	2	3	2	2	**2**
COD	245	245	246	246	245	243	245	245	245.7	245	**243**
Turbidity	5	5	5	4	3	−	3	4	3	4	**3**

The locations in Table 6, the second column, are the most critical regarding the exceedance margin from the threshold values. These results were obtained by applying Eq. (1) to Eq. (7) for those physical and chemical water quality parameters that exceed the threshold value. It is noted that Bara River's water quality parameters behaved differently among all locations of parameter values. The table below confirms the identification of Bara River as the most critical location in at least seven water quality parameters. All these locations exceed the threshold values by considerable margins. At the same time, the locations identified in the third column are the most vulnerable due to applying Eqs. (8) and (9) for the physical and chemical water quality parameters that nearly exceed the threshold value. These results closely match the physical reality on the ground. The Khairabad station appears the most in Table 6, which is a location where the Kabul River, along with all its tributaries, meets the Indus River. The water quality parameters, for example, TSS, Na, TH, Mg, and SO_4_^2-^ are on the upper side of allowable limits. The location is vulnerable because further deterioration will change these locations to critical areas. Adezai, Jindi, and Pabbi Rivers and Warsak Dam appear twice in Table 6; all these locations inhabit and sustain large population densities along their banks. In addition to agriculture, small-scale industries (Ghee, Marble, Paper industries, and Auto-Repair Shops) are predominantly the means of sustenance for the local population.

**Table 6:** Critical location and vulnerable locations are based on Tables 3, 4 and 5.

Parameters	Critical location (CL)	Vulnerable location (VL)
Total suspended solids	BR	AK
Electric conductivity	PR	WD
Dissolved oxygen	RR	AK
Biochemical oxygen demand	BR	PR, JR, RR
Turbidity	BR	PR, JR
Total alkalinity	BR	PR
Sodium	BR	IR
Calcium	AK	NK
Total hardness	BR	IR
Magnesium	BR	IR
Nitrite	NK	NG
Total dissolved solids		IR
Chemical oxygen demand		BR
Sulfate		BR
Chloride		WD
Phosphate		RR
Arsenic		JR

**Figure 8:**
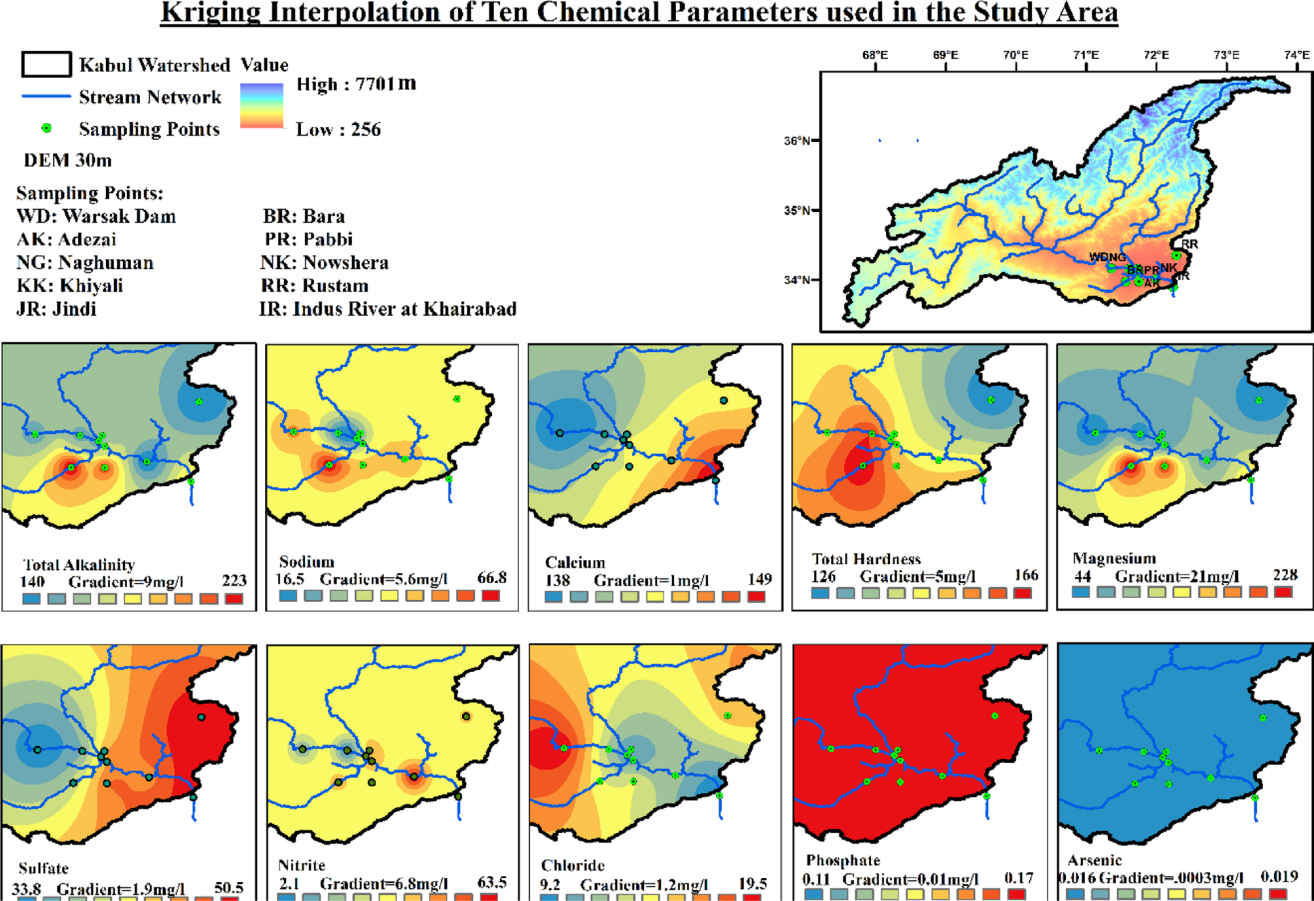
Interpolated values of water quality physical parameters over the entire study area.

**Figure 9:**
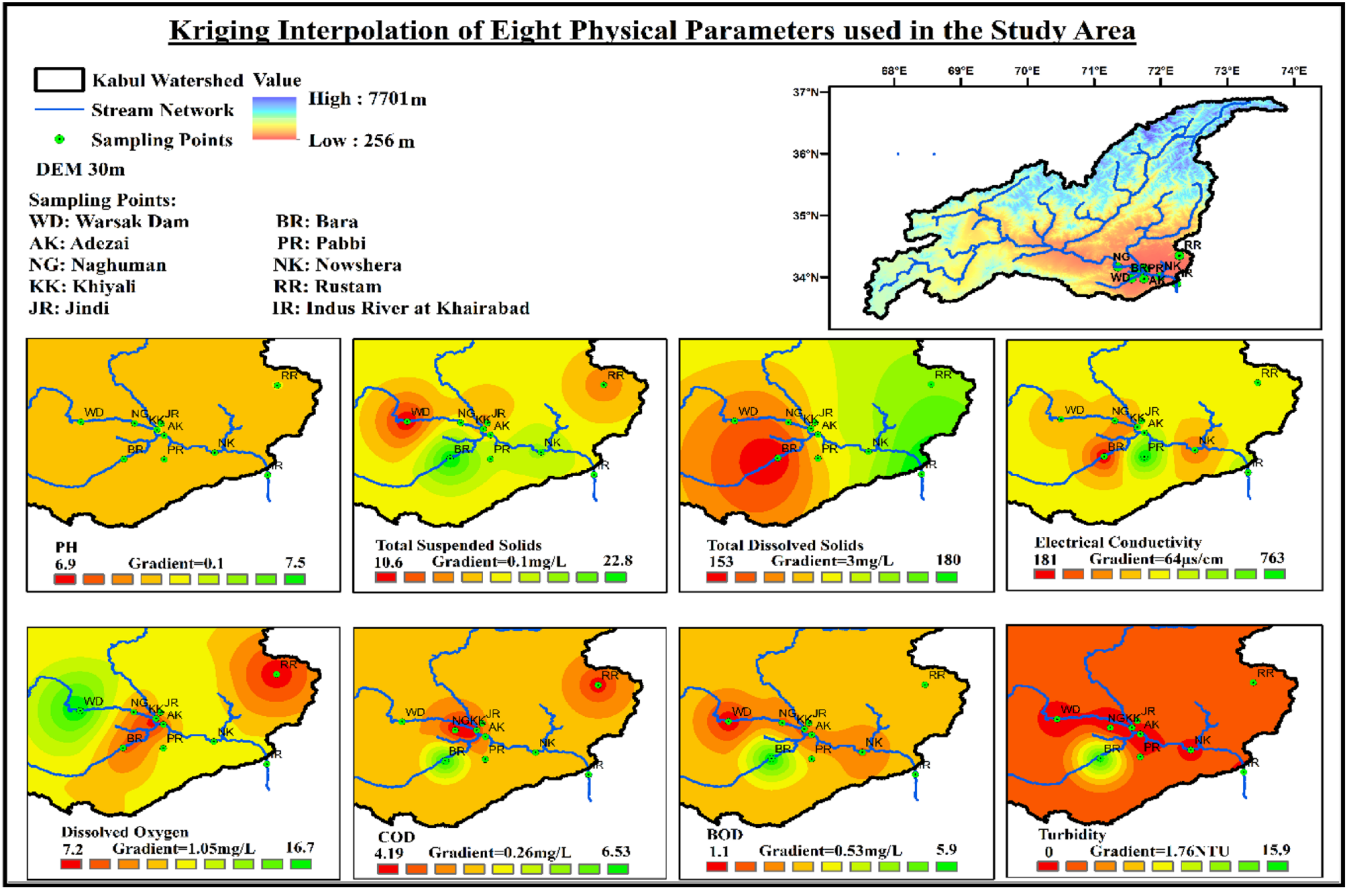
Interpolated values of water quality chemical parameters over the entire study area.

**Figure 10:**
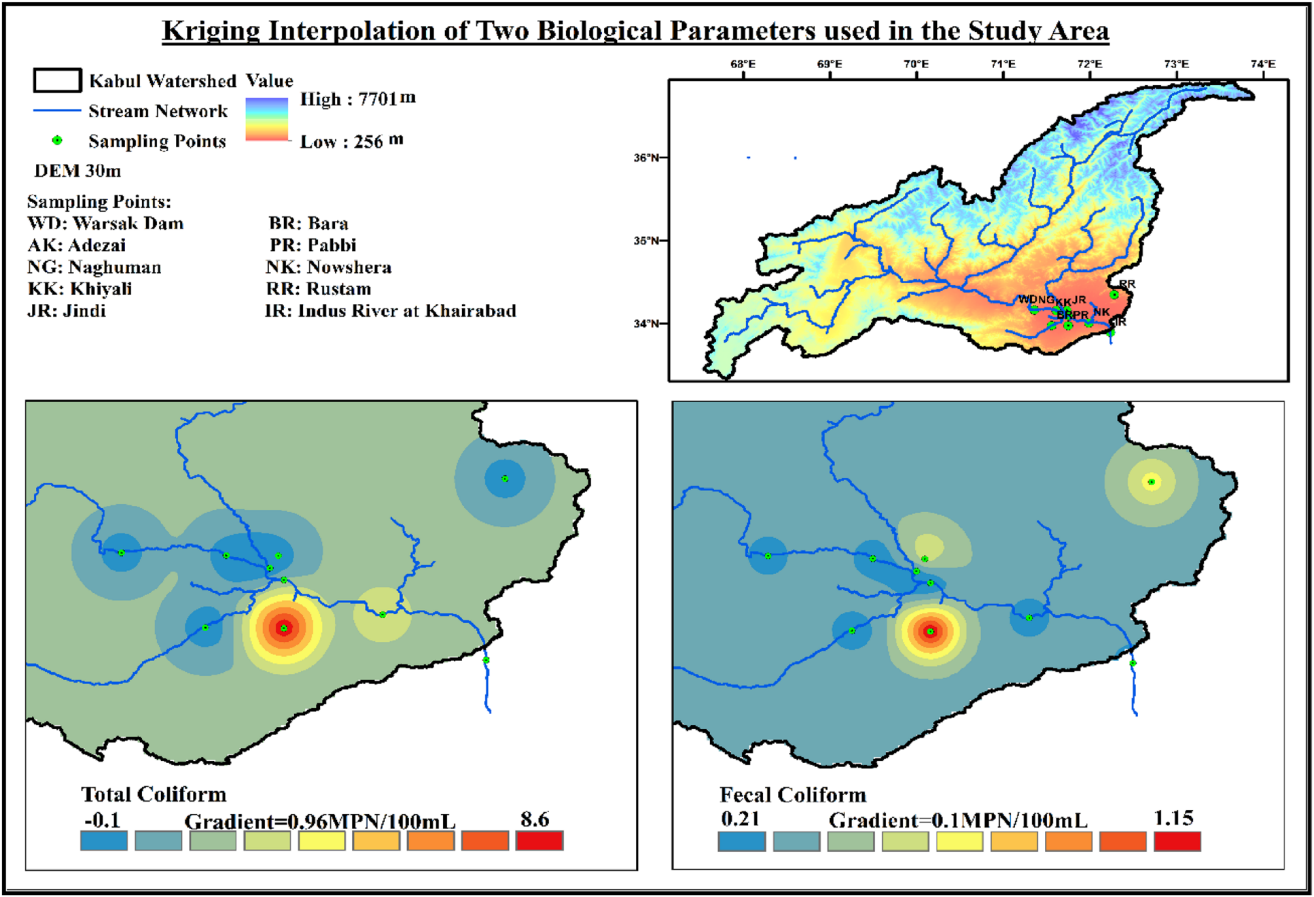
Interpolated values of water quality biological parameters over the entire study area.

The method mentioned in “Vulnerable Location Identification” Section was employed to compare the results obtained for critical and vulnerable locations. Figures 8, 9, 10 shows the areas identified by kriging interpolation as the most critical ones. The areas are mostly the same identified through the distance, and weightage-based identification method explained above. Bara River is in the most critical state due to the large margin exceeding the threshold values of different parameters. Mitigating and preventive measures must be taken at the identified location to improve the water quality parameters, especially TSS, BOD, Turbidity, TA, Na, TH, and Mg.

## Discussion

The study utilized above mentioned mathematical equations to analyze the water quality of the Kabul River using distance and weightage-based methods to highlight the most critical and vulnerable locations. The analysis of the results presented in Table-3 revealed that multiple water quality parameters across different locations exceeded the escalated values, except for dissolved oxygen. The Bara River (BR) had the highest number of exceedances, followed by PR, RR, JR, IR and AK, as highlighted in Table 3. Table 6 further supported this observation by identifying BR as the most critical location in at least seven water quality parameters. Table 5 identified vulnerable values in each row and indicated the most vulnerable locations. The Khairabad station, located at the confluence of the Kabul River and its tributaries with the Indus River, frequently appeared in Table 6 and exhibited water quality parameters on the upper side of allowable limits. The consistency between the results obtained through different methods further validated the findings. 

## Conclusion

The study developed two objective functions applied to different locations in the larger prone area of Peshawar District, Pakistan. The methodology identified the critical areas and vulnerable locations concerning the water quality part. The results show considerable skills in identifying the critical and vulnerable locations compared with already published work for the same area of water quality parameters. The developed method is intuited and easy to apply and gives results, which are enclosed tendons with the ground reality of the region. The study employed mathematical Eqs. (1–9) to assess water quality parameters in various locations, revealing that the Bara River had the highest number of exceeded values, signifying its critical condition.

Adezai, Jindi, Pabbi Rivers, Khairabad station, and Warsak Dam were also vulnerable due to parameters nearing the threshold values. These findings correspond to the actual state of the water bodies. Effective measures should be implemented to safeguard these rivers, targeting the improvement of crucial parameters such as Total Suspended Solids, BOD, Turbidity, Total Alkalinity, Sodium, Total Hardness, and Magnesium. These protection measures could involve implementing pollution control measures, enhancing wastewater treatment facilities, promoting sustainable agricultural practices, and raising awareness among local communities about the importance of preserving water quality. Ensuring the long-term health of these rivers requires a collaborative effort from stakeholders, including environmental organizations, government authorities, and local communities. By implementing these measures, we can move towards restoring and maintaining these vital water resources' ecological integrity and sustainability for both present and future generations.

## Data Availability

The data generated and analyzed during the current study are included in this article. The analyzed data in the form of an Excel sheet is attached in the supplementary files.
